# Improvdent: Improving dentures for patient benefit. A crossover randomised clinical trial comparing impression materials for complete dentures

**DOI:** 10.1186/1472-6831-12-37

**Published:** 2012-08-31

**Authors:** Janine C Gray, Nuria Navarro-Coy, Sue H Pavitt, Claire Hulme, Mary Godfrey, Helen L Craddock, Paul A Brunton, Sarah Brown, Sean Dillon, Gillian Dukanovic, Catherine Fernandez, Jonathan Wright, Howard Collier, Shirley Swithenbank, Carol Lee, T Paul Hyde

**Affiliations:** 1 Clinical Trials Research Unit, University of Leeds, Leeds, LS2 9JT, UK; 2 Centre for Health Sciences Research, Leeds Institute of Health Sciences, University of Leeds, Charles Thackrah, 101 Clarendon Road, Leeds, LS2 9LJ, UK; 3 Academic Unit of Health Economics, Leeds Institute of Health Sciences, University of Leeds, Charles Thackrah, 101 Clarendon Road, Leeds, LS2 9LJ, UK; 4 Centre for Health and Social Care, Leeds Institute of Health Sciences, University of Leeds, Charles Thackrah, 101 Clarendon Rd., Leeds, LS2 9LJ, UK; 5 Leeds Dental Institute, Clarendon Road, University of Leeds, Leeds, LS2 9LU, UK; 6 IMPROVDENT PPI representatives, c/o Leeds Dental Institute, Clarendon Road, University of Leeds, Leeds, LS2 9LU, UK; 7 Dental Translational and Clinical Research Unit (DenTCRU), Leeds Dental Institute, Clarendon Road, University of Leeds, Leeds, LS2 9LU, UK

**Keywords:** Complete dentures, Edentulous, Impression material, Cost-effectiveness, Crossover trial

## Abstract

**Background:**

According to the UK Adult Dental Health Survey (2009) 15% of adults aged 65–74, 30% aged 75–84 and 47% aged >85 years are edentulous and require complete dentures. Patients’ quality of life and nutrition status are affected by poor dentures. The quality of the dental impression is the most important issue for improving the fit and comfort of new dentures. There is paucity of RCT evidence for which impression material is best for complete dentures construction. This study aims to compare two impression materials for effectiveness and cost effectiveness.

**Methods/Design:**

IMPROVDENT is a double-blind crossover trial comparing the use of alginate and silicone, two commonly used denture impression materials, in terms of patient preference and cost-effectiveness. Eighty five edentulous patients will be recruited and provided with two sets of dentures, similar in all aspects except for the impression material used (alginate or silicone). Patients will try both sets of dentures for a two-week period, unadjusted, to become accustomed to the feel of the new dentures (habituation period). Patients will then wear each set of dentures for a period of 8 weeks (in random order) during which time the dentures will be adjusted for optimum comfort. Finally, patients will be given both sets of dentures for a further two weeks to wear whichever denture they prefer (confirmation period).

Patients will be asked about quality of life and to rate dentures on function and comfort at the end of each trial period and asked which set they prefer at the end of the habituation period (unadjusted denture preference) and confirmation period (adjusted denture preference). A health economic evaluation will estimate incremental cost-effectiveness ratios of producing dentures from the two materials. A qualitative study will investigate the impact of dentures on behaviour and quality of life.

Funding: IMPROVDENT is funded by NIHR RfPB (PB-PG-0408-16300).

**Discussion:**

This trial aims to provide evidence on the costs and quality of dentures cast from two different commonly used impression materials; the intention is to significantly impact on the quality of denture production within NHS dentistry.

**Trial Registration:**

ISRCTN Register: ISRCTN01528038

UKCRN Portfolio ID: 8305

## Background

According to the UK Adult Dental Health Survey (2009) one in five adults wear dentures and 6-10% of all UK adults were edentulous rising to 15% of adults aged 65–74, 30% aged 75–84 and 47% aged >85 years [1]. The epidemiology demonstrates an uneven distribution of edentulous patients when they are defined by age, sex, socio-economic class, and geographical area of residence. Broadly, the older the patients are in the sample the higher the number of edentulous patients, the lower the socio-economic status the higher the edentulous rate, there are more women than men who are edentulous and the prevalence in England is higher in the Midlands and North compared to the South and East. These are patients who we can expect to rely on the NHS to provide dental treatment. The dentures with which they are provided have an impact on their quality of life and nutritional status.

There are published studies which have looked at the impact of tooth loss on the Oral Health Related Quality of Life (OHRQoL) using a number of different assessment tools. Jagger et al [2] found that ”Denture related problems had a negative impact on quality of life of both partially dentate and elderly patients”. Heydecke et al [3] say “Wearing conventional complete dentures has a significant {negative} impact on OHRQOL”. John et al [4] conclude “Denture status was a stronger predictor for impaired OHRQoL than demographic variables and rendered age and education almost negligible in their influence on OHRQoL”. Adam et al [5] conclude “This study shows that after the provision of a new set of complete dentures the OHRQoL of patients improved significantly”. Ellis et al 2007 [6] state “In this study, the provision of new dentures either with a conventional technique or with a duplication technique resulted in an overall improvement in oral health-related quality of life and satisfaction”. Overall the literature shows both a significant negative impact of (old) dentures on a patients quality of life and an improvement in life quality when the patients are provided with new dentures. The literature on nutritional problems associated with the edentulous state is summarised by Ellis et al 2008 [7], who found that the literature suggested that impairment of masticatory function may lead the edentate to adapt their food choices to avoid foods that they find difficult, alter food preparation in order to cope with their masticatory inefficiencies or to swallow partially masticated food. They found that the literature further suggested that dietary limitations and inadequacies (a diet high in calories and fat, low in wholemeal bread, cereals, fruit and vegetables) may result in the edentulous having demonstrable nutritional deficiencies when compared with a similarly aged dentate population, resulting in poorer health [7]. Provision of new dentures showed an improvement in the nutritional choices available to the patients [7].

In an aging population the nutritional status and the quality of life of edentate individuals can be improved by the provision of good quality dentures. Experts in prosthodontics concur that the quality of the dental impression is the most important issue for improving the fit and comfort of a new denture. The choice of material for the crucial impression is important but there is paucity of evidence for which impression material is best for complete denture construction. Harwood [8] points out that there have only been 5 randomised controlled trials (RCT) reported which looked at the choice of materials for dental impressions. Only 2 of these 5 studies looked at impressions for complete dentures (i.e. of mucosa alone). These 2 studies [9,10] were inconclusive and they did not include the impression material used by 95% of UK dentists namely alginate [11].

Petrie [12] shows that among experienced USA dentists (ACP Members) the single most used impression material was polyvinyl siloxane (silicone). The Petrie [12] and Hyde [11] papers show a dichotomy of transatlantic opinion and practice. This proposal is for a randomised controlled trial (RCT to compare the 2 impression materials (alginate and silicone) for effectiveness and cost effectiveness, and fill the evidence gap for best practice.

## Methods

### Trial objectives

#### Primary objectives

•To establish whether there is a patient preference for unadjusted dentures produced from alginate or silicone impressions.

•To assess cost-effectiveness for dentures produced from alginate versus silicone impressions.

#### Secondary objectives

•To assess the impact of dentures produced from alginate and silicone impressions on oral health related quality of life.

•To assess comfort, mobility and chewing efficiency for dentures produced from alginate or silicone impressions.

•To assess the patients’ experience of having impressions made using alginate and silicone impression materials.

•To assess by qualitative research methodology, the impact of the dentures on patient perceived life quality.

## Design

IMPROVDENT is a single centre, randomised crossover study. This design is preferred because previous high quality studies have been successful in differentiating individual preferences for different types of prosthesis [13-15]. For each patient the aim is to produce 2 sets of dentures which are similar apart from the impression material used to mould the fitting surface. Patients will assess the comfort, mobility and chewing efficiency of each set of dentures: the patients will be asked to state which is their preferred set of dentures.

### Recruitment

Over a 22 month period 85 edentulous patients will be recruited from the Leeds Dental Institute’s (LDI) waiting list for replacement of complete dentures and from local general dental practitioners.

Patients will be approached during standard clinic visits and will be provided with verbal and written details about the study (Patient Information Sheet and Informed Consent Document). This will include detailed information about the rationale, design and personal implications of the study.

Alternatively, patients identified by other means (such as waiting lists or review of case records) may be sent a personalised letter inviting them to take part. This letter will include a brief introduction to the study. Patients will be invited to contact the research team at the LDI to find out more information and to make an appointment to discuss the study further.

In addition to the recruitment process described above, it has been recognised that if recruitment rates from the patient waiting lists at the LDI are insufficient, then recruitment of suitable patients directly from General Dental Practice (GDP) would be beneficial. General dental practitioners will be sent an invitation letter inviting them to identify potentially suitable patients for the study. They will also receive a summary outlining the nature of the study and details of the inclusion/exclusions to identify suitable patients. General dental practitioners (GDPs) will ask suitable patients if they wish to be considered for potential inclusion into the study. Interested patients will be given a patient leaflet with a brief summary about the study and details of how to contact the research team. When a patient contacts the research team they will be invited for assessment and if suitable and willing recruited to the study using the same procedure described above for LDI patients.

Patients recruited from the LDI waiting lists will generally be referred by GDPs as more complex cases for full denture construction, e.g. patients who have worn dentures for several decades and have a seriously resorbed mandible. In contrast patients recruited directly from GDPs may not have such severe resorption and might otherwise have been treated at their own dental practice.

Following information provision, patients will have as long as they need to consider participation (at least 24 hours) and will be given the opportunity to discuss the study with their family and other healthcare professionals before they are asked whether they would be willing to take part in the study. Assenting patients will then be formally assessed for eligibility and invited to provide informed, written consent.

#### Inclusion criteria

1.Patients who are edentulous.

2.Patient is available for follow up.

3.Patient requires replacement complete dentures.

4.Patient is able and willing to complete the informed consent process.

5.Age 18 years or older at the time of signing the Informed Consent Form.

#### Exclusion criteria

1.Presence of an oral tumour.

2.Requirement for an obturator.

3.Extreme xerostomia (e.g. Sjögren’s syndrome).

4.Patients who would benefit from selective pressure impressions.

5.Known hypersensitivity to silicone or alginate.

### Randomisation and blinding

A series of randomisations and colour coding of the dentures will be introduced to ensure patients, dentists and dental nurses will be unaware of the impression material used to construct each set of dentures and the order of testing each denture made from each impression material. Dentures will be coded using a small coloured dot for identification – red or blue during the habituation period and green or yellow during the adjustment periods. It is likely that patients will form a preference for a particular set of dentures during the habituation period; dentures are re-coded after the habituation period in order that patients are unaware of which denture they are being given during the adjustment periods.

The Chief Investigator will carry out the clinical stages of denture construction up to and including the delivery of the dentures. The visits for the adjustment and assessment of the dentures will be conducted by an independent Consultant in Restorative Dentistry. The assessments of denture preference are patient reported.

**Figure 1:**
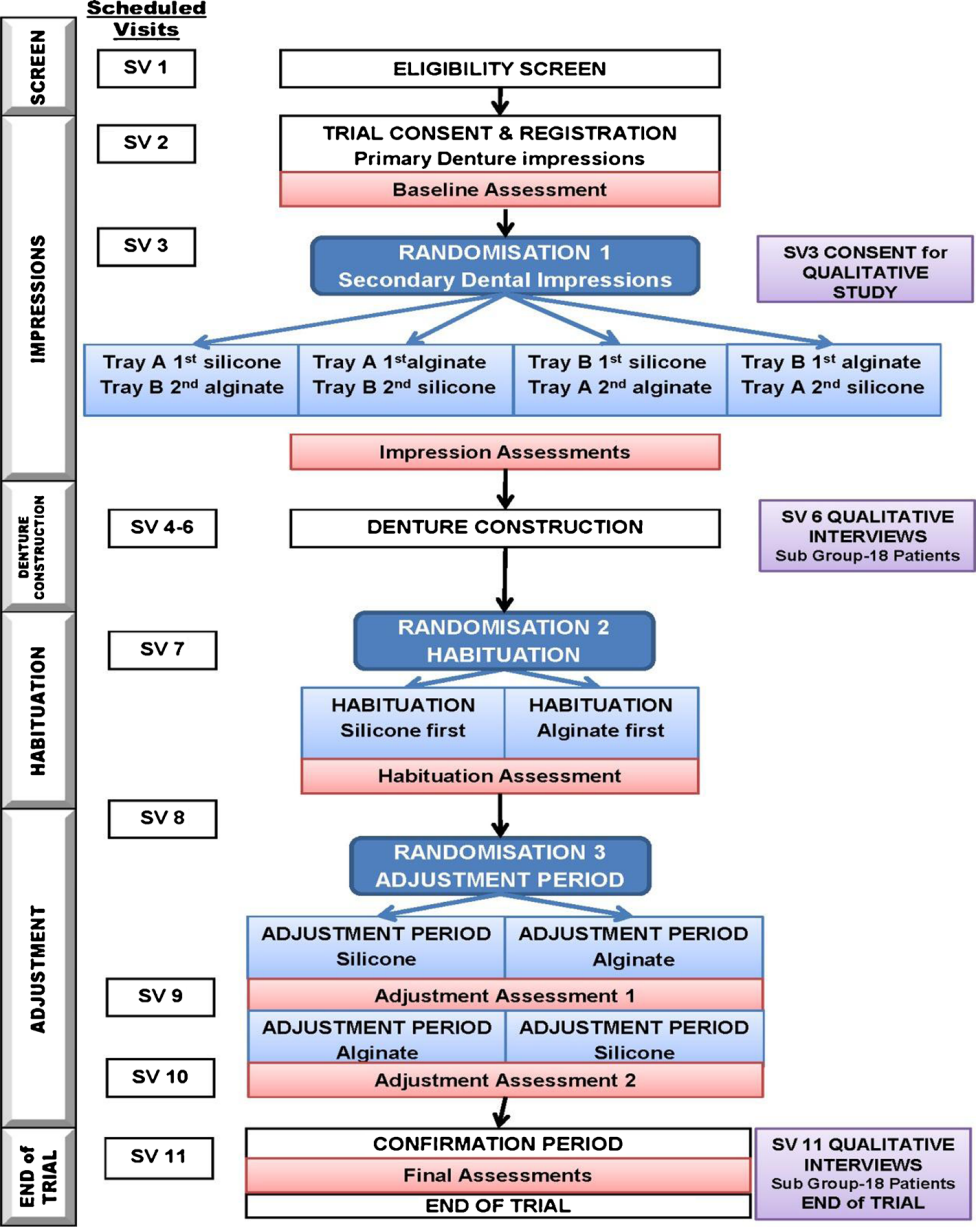
IMPROVDENT Trial Flow Diagram.

Patients will undergo three randomisations (refer to Figure 1):

The initial randomisation occurs at the baseline visit using sealed envelopes to determine which tray is used to make the first impression and which impression material is used first (silicone or alginate). Envelopes are created by the trial statistician, using random block sizes, and are stored in a secure randomisation locker. The habituation randomisation occurs at the completion of denture construction to determine the colour marking (red or blue) of the dentures (silicone red/alginate blue or alginate red/silicone blue, the red denture to be tested first during habituation period). Randomisation will be via sealed envelopes created by the trial statistician. Randomisation will be blocked (random block size) to ensure balance between groups. Sealed envelopes will be stored in the secure randomisation locker.

The aim of the adjustment randomisation is to establish the order of testing during adjustment. This final randomisation will occur at the conclusion of the habituation period to determine the colour re-marking (yellow or green) of the dentures (silicone yellow/alginate green, alginate yellow/silicone green, yellow to be tested in adjustment period 1 and green to be tested in adjustment period 2). Randomisation will be blocked (random block sizes) and balanced for order of testing in the habituation period. Randomisation will be administered by telephone by CTRU, using an automated 24-hour telephone system.

### Denture construction

The Prosthodontic Research Team at LDI has developed and published a method of producing two or more sets of dentures which are clinically similar apart from the one deliberate alteration which is to be the subject of an RCT [16]. This methodology will be used to produce two sets of dentures which are clinically similar apart from the impression material that was used to provide the mould for the fitting surface of each denture.

The intervention at the clinical secondary impression stage is the subject of this study; the secondary impressions will be made with either alginate or silicone. To take the secondary impressions, two sets of similar, spaced (2 mm), unperforated, light-cured acrylic secondary impression trays will be constructed. A set of impression trays consist of an upper and a lower tray; each set will be numbered in the laboratory A or B. Clinically, the secondary impression trays will be trimmed to size in the mouth to remove any over extension. After trimming, the trays will be randomly allocated to either the alginate or silicone impressions. The impression trays allocated to the alginate side of the study will be ‘border moulded’ with greenstick compound in the standard way [17] and alginate (Xantalgin®) impressions taken. The trays allocated to silicone will be ‘border moulded’ with silicone (express 3 M) and impressions taken with silicone (express 3 M). The order in which the impressions are taken will be randomised; the patients will be rested for comfort for approximately 30 minutes between taking the first set of impressions and taking the second set of impressions. If a clinically adequate impression is not achieved at the first attempt with either material the impression will be repeated, until clinical adequacy is achieved. The total time taken to make the impression and number of attempts will be recorded.

Both sets of impressions will be immediately rinsed and disinfected using a proprietary disinfectant. After disinfection the alginate impressions will be wrapped in damp gauze and sealed in an airtight plastic bag; this is to comply with normal practice and the alginate manufacturer’s instructions. The silicone will be shaken dry and then placed in a plastic bag. The following day, both sets of impressions will be checked and cast with vacuum mixed dental stone. Once cast, the models produced will be thoroughly cleaned to remove all traces of alginate or silicone impression material. The models from the different impression materials will be permanently numbered by the Head of the Prosthodontic Research Laboratory (or an individual authorised to do so by the Chief Investigator) according to a randomisation schedule provided by the trial statistician. All dentists will be blind to which set of models came from which impression material.

In order to ensure similar dentures are constructed for both impression materials, the protocol for occlusal registration and wax trial insertion in the paper by Dillon et al [16] will be followed. This protocol utilises silicone moulds of the occlusal and polished surfaces to shape and position teeth and surfaces of the duplicate denture. The dentures will be constructed with balanced occlusion and where possible given a balanced articulation on an average value articulator. Once construction of both sets of dentures is complete, the sets will be marked with a red or blue acrylic dot according to the sealed envelope containing the random allocation provided by the trial statistician, to randomise the order of wearing during the habituation period. Insertion and delivery of the completed dentures will proceed in the usual way.

### Habituation period (2 weeks)

Once dentures have been constructed, patients will be given both sets of dentures for a 2-week habituation period. The habituation period serves a dual purpose: firstly to allow patients to try both sets of unadjusted dentures to establish whether one set is preferred. Secondly, it has been suggested in a previous crossover study [19] that patients tend to prefer the denture given in the second period because the first period is spent habituating to the feel of the new dentures which may feel very different to their old dentures. Thus by allowing a habituation period in this study it is hoped that patients will be used to the feel of the new dentures prior to the two adjustment study periods. During the 2 weeks the patients will be provided with a diary and asked to record the wearing of each set of dentures. Within the diary there is a randomised and structured programme alternating the use of the red and blue dentures with an opportunity for patients to provide qualitative feedback regarding the impact on behaviour and interactions in social settings wearing the new dentures. On the first day, they will be asked to wear the red denture; on the second day the blue denture. This initial period of alternation is to avoid familiarity and habituation clouding the later assessment. After 2 days they will be guided to wear the red-coded denture for 3 days followed by the blue-coded denture for 3 days. They will then be asked to frequently alternate the dentures for 2 days. Finally, for the last 4 days of the 2-week habituation period, they will be asked to wear whichever denture they prefer. On return to clinic they will be asked to report their preferred denture. If they have no preference they will be asked if this is because they were satisfied by both sets of dentures or if it is because both sets are unsatisfactory. They will then be asked to score each denture for comfort, mobility and chewing efficiency using a 5-point Likert scale and will complete a health economics questionnaire. Both sets of dentures will be returned and re-marked with a yellow or green spot by the Head of the Prosthodontic Research Laboratory (or designee).

### Adjustment period 1 (8 weeks)

The yellow set of dentures will be returned to the patient first. If any adjustment or remedial work is required to make the dentures comfortable, retentive and useful, this will be carried out by an experienced clinician. Within the limits of the LDI appointment system there will be no restriction on the number of appointments the patients can request during this 8 week period. Every appointment requested by the patient will be noted and a detailed record of the work required will be kept. The cost of any remedial treatment will be calculated. Although there is provision in this study for the adjustment of the dentures, it is expected that the majority of patients will require little adjustment. At the end of this first 8 weeks the patient will be asked to attend and report the impact the dentures have had on their quality of life by the completion of OHIP-EDENT and EQ-5D questionnaires and will rate the denture for comfort, mobility and chewing efficiency using 5-point Likert scales. Patients will also be asked to complete a health economics questionnaire.

The denture will be returned to the clinic and the green set of dentures given to the patient.

### Adjustment period 2 (8 weeks)

Any adjustments required for the second (green) set of dentures will be made and recorded as for Adjustment Period 1 (yellow). After 8 weeks the patient will return to clinic for a final assessment and asked to complete the OHIP–EDENT, EQ-5D and health economics questionnaires and will rate the denture for comfort, mobility and chewing efficiency. A sub set of patients will also be asked to think back to the two experiences they have had with the fitting/adjustment/wearing of the yellow (adjustment period 1) and then the green denture (adjustment period 2) and express any preference that they may have.

### Confirmation period (2 weeks)

After the two 8 week periods, it is to be expected that each set of dentures will be as good as it is possible to make them. The patient will be asked to take both sets of dentures away for a 2-week confirmation period. During this confirmation period the patient will be able to use whichever dentures they wish. They will be asked to return for a final visit to express any preference for the dentures and perform a Likert assessment of comfort, mobility, and chewing efficiency.

### Outcome measures

The co-primary outcome measures of the trial are patient expressed preference for unadjusted dentures and cost-effectiveness.

Secondary outcome measures are patient preference for adjusted dentures, patient assessment of comfort and taste (using a 5-point Likert scale) and preference of the impression materials, patient assessment of comfort, mobility, chewing efficiency of the dentures (using 5-point Likert scales).

Patient preference for dentures will be established by asking whether one or other denture is preferred, or whether there is no preference. Those expressing no preference will be further asked whether both dentures were satisfactory or unsatisfactory. Patients will be asked to reflect on the whole process of receiving the final denture (including initial comfort, amount of adjustment needed) when expressing preference for the adjusted denture.

Health-related quality of life will be measured using the OHIP-EDENT questionnaire [18]. This questionnaire is adapted from the Oral Health Impact Profile (OHIP) and is particularly suitable for edentulous patients.

### Assessments

Preference will be assessed for all patients at two time points during the trial. Patients will be asked to express a preference for the unadjusted dentures at the end of the habituation period, and preference for adjusted dentures at the end of the confirmation period. A sub set of patients will be asked to express a preference for the adjusted dentures at the end of the second adjustment period.

OHIP-EDENT and EQ-5D will be measured at baseline and at the end of adjustment periods 1 and 2.

A health economics assessment questionnaire will be completed at the end of the habituation period, and at the end of adjustment periods 1 and 2.

Assessment of comfort, taste and preference for the impression materials will be made immediately after both sets of impressions have been taken.

Assessment of Comfort, Mobility, and Chewing Efficiency will be assessed at the end of the habituation period, each adjustment period and the confirmation period.

A qualitative interview will be conducted on a subset of 18 patients in the course of scheduled visits when the dentures are being constructed and after the end of the confirmation period.

### Sample size

A sample size of 76 patients would have 80% power to detect a difference in preference rates of 30% between the two dentures (30% vs 60%) at a significance level of 5%, assuming that 10% of patients express no preference; 67 patients would be required to detect a different of 30% (25% vs 55%) assuming 20% express no preference. A total of 85 patients will be recruited overall to allow for a dropout rate of around 10%, consistent with previous studies.

### Statistical analysis

Preference results will be presented as a 2 × 2 table for paired data and analysed using McNemar’s test for paired data to estimate the difference in proportions of patients preferring dentures made from alginate impressions compared to those made from silicone impressions, prior to any denture adjustment. Those patients expressing a preference will be allocated to the discordant cells of the table and those expressing no preference will be allocated to the concordant cells according to whether they felt that both sets of dentures were either satisfactory or unsatisfactory. The difference in proportions will be presented with a 95% confidence interval.

OHIP–EDENT scores at the end of each adjustment period will be analysed using an ANOVA model appropriate for an AB/BA crossover design. The model will incorporate denture, period, and subject, all as fixed effects. The difference between the dentures will be estimated with 95% confidence intervals.

Differences between Likert scores (measuring comfort, mobility, chewing efficiency, comfort of impression taking, taste and preference of impression taking) for each denture will be calculated and compared using the Wilcoxon test for matched pairs.

All analyses will be conducted on the intention-to-treat (ITT) population where patients will be analysed according to the treatment they were randomised to, and the per-protocol population (if necessary), where patients will be included according to the treatment they received.

An additional analysis will test whether the differences in comfort, mobility and chewing efficiency between the two dentures varies according to the timing of the assessment (i.e. between the adjustment and confirmation periods). The 5-point Likert questionnaires are completed after adjustment period 1 (wearing the yellow denture), and also at the end of adjustment period 2 (wearing the green denture). The confirmation period requires patients to complete the 5-point Likert questionnaire side-by-side after wearing whichever denture they preferred during the 2-week period. Wilcoxon tests for matched pairs will be performed on the difference in scores between the two dentures in the adjustment and confirmation periods.

Only patients who wore and completed the assessment for both dentures in the confirmation period will be included in this analysis.

## Economic evaluation

### Cost effectiveness

The primary objective of the economic evaluation is to identify the within study incremental cost effectiveness ratios; the costs and benefits of the use of silicone compared to the costs and benefits of alginate over the duration of the study. Use of incremental cost effectiveness ratios will enable comparison of the additional financial costs imposed use of silicone over alginate with any additional benefits it delivers.

The primary analyses will take the perspective of the service provider including the costs of health and social care. In line with the clinical study patient data will be collected over the course of the dental construction and assessment period. The within-study analysis will estimate the expected incremental cost per point decrease in OHIP-EDENT score. In addition a secondary analysis will use quality adjusted life years (QALYs) outcome measures. The estimation of QALYs requires the production of utility weights for each health state observed in the study population. We will use the EQ-5D (Euroqol) instrument for this purpose [19,20]. The EQ-5D is a very simple instrument to complete and will be collected at the same time and using the same methods as the other outcome data. This will limit the need to interpolate quality of life between observation points and the associated inaccuracy in the estimation of the health related quality of life differences between the interventions.

Health resource use associated with each treatment modality will be collected during each dental visit to contribute to a health economics analysis of additional health financial costs related to treatment and the study. This will include the service provision together with use of community and hospital based health care attributed to the treatment. Community and hospital based health care will be collected by way of a short patient questionnaire completed in the clinic. The patient questionnaire will be designed to allow tick box completion wherever possible. Unit financial costs for health services resources will be obtained from national sources such as the PSSRU, the BNF and NHS reference cost database.

Whilst the primary analyses will adopt the perspective of the NHS and social services, secondary analysis will adopt a societal perspective taking account of productivity costs and out of pocket expenditures incurred by the patients. The patient questionnaire will also be used to collect this data (for example, travel expenses). The data for collecting the indirect financial costs associated with each intervention (for example, time away from work) will also be collected through the patient questionnaire. There is currently uncertainty about the best method for estimating productivity costs; we will adopt the recommended method.

### Analysis

The incremental cost effectiveness ratios will be calculated as the difference between the mean costs and difference in OHIP-EDENT score/QALYs in each arm. The non-parametric bootstrap method will be used to produce a within study probabilistic sensitivity analysis of the incremental cost effectiveness ratios. The expected incremental cost effectiveness ratio, a scatter plot on the cost effectiveness plane, the 95% cost effectiveness eclipse and the cost effectiveness acceptability curve will be presented. Given the duration of the study discounting is not required.

## Qualitative study

The objective of the qualitative component of the study is to provide, from the patient perspective an in-depth understanding of expectations around treatment, treatment preferences and user centred outcomes. These are intended to complement the use of standardised tools (Likert Scales and Oral Quality of Life Questionnaire). They will be conducted at two points in time: in the course of scheduled visits to the dentist when the new dentures are being constructed, and at final assessment of the new adjusted dentures, at the end of the study.

### Sampling strategy

From among those consenting to be interviewed, a purposive sample of 18 patients will be initially selected to reflect dimensions of interest in terms of experience of tooth loss and expectations of treatment: age (under 40 years; between 41 and 60 years; 61 + years); gender; length of time with a full set of dentures (under 10 years; 11–20 years; 21 years and over); and socio-economic circumstances (housing tenure and 4-digit postcode. The sample size is sufficiently small to facilitate the collection of ‘information rich’ data (in-depth, detailed and nuanced); whilst being large enough to include patients whose experiences may differ in patterned ways. Thus the findings will enable insight into the social contexts that shape perceptions, meanings and experiences, complementing and extending those drawn from use of structured data collection methods and statistical analysis. We will, however, review the sample size in light of the data obtained leaving open the possibility of recruiting up to five further participants if it appears that themes emerging from the completed interviews require further exploration.

### Data collection

Using a loose topic guide, the first set of interviews are aimed at developing understanding about the meaning and significance of tooth loss, its course over time, the strategies employed to manage it, accessing treatment, treatment choices, preferences and expectations about outcomes. We will explore how tooth loss and denture related problems have impacted over time on patients’ behaviour in both public and private domains as they go about their daily lives; and affected social relationships, leisure pursuits, social activities, diet and eating pattern. We will examine impact on self-esteem and sense of self and the coping strategies people have employed to manage the difficulties encountered. The interviews will also examine what patients would most value as an outcome of good fitting dentures; their expectations about the kinds of changes they anticipate and what changes they would most value. A key difficulty in any assessment of life quality is that it is bound up with individual expectations. Yet expectations may be altered as a result of changing circumstances – whether lowered or heightened – posing a problem of measurement of quality of life. Developing an in-depth picture of experiences, behaviour, coping strategies and expectations relating to edentulous status will offer a clearer understanding of how and in what ways the new dentures affect those dimensions of life quality that are important to patients with different characteristics.

These qualitative interviews then will be guided by a list of topics to be covered rather than a set of pre-determined questions. The intention is to provide patients with the opportunity to talk about things that are salient to them and at the pace dictated by them within the framework provided by the topic guide. It is anticipated that interviews will last around an hour. Their content and structure will be discussed with our patient and older people’s reference/advisory groups.

A second set of interviews will be carried out with these patients at the end of the final assessment of their new adjusted dentures. We will examine patients’ experience of the intervention, what factors drove their choice of denture, any difficulties they might have had in stating a preference. We will also examine the relationship between prior expectations and actual experience with the new dentures including changes that may have occurred in behaviour, social relationships, social activities, diet and eating patterns; and if and in what ways the new dentures have made a difference to those dimensions of life quality on which they place high value. We anticipate that these interviews will also last around an hour. Topic guide for both interviews will be refined through involvement of our PPI representatives and older members of Caring Together that comprise our reference group, in a workshop organised prior to the commencement of the study.

### Data analysis

All of the interviews will be audio-taped, fully transcribed and analysed using a computer assisted qualitative analysis package. A grounded theory approach to analysis will be employed [21], proceeding through the interplay between induction and deduction. Themes and patterns will be identified through open coding and categories and their constituent properties will be developed through comparing and contrasting coded segments. Of particular interest is the extent to which such factors as age, gender, length of time without teeth and socio-economic status impact on user centred outcomes and quality criteria and preferences.

## Discussion

Prosthodontic research has historically had a lack of RCT evidence [8]. RCTs published in the prosthodontics literature have been reported poorly, lacking details regarding the details of randomisation and blinding which calls into question the quality of the studies themselves [22]. This indictment of academic prosthodontics was a challenge for future researchers.

Carlsson looked at the lack of RCT evidence and went on to suggest that it is “not probable that comparisons between dentures made with varying materials and methods would lead to significant differences”. However the opinion of the authors is different; we believe the reasons for the lack of success with denture RCTs is the quality of protocol design coupled with twin problems within prosthodontics of multiple confounding variables and a sensitive meaningful outcome measure. The authors have accepted the challenge implied by Jokstad to design and execute a productive prosthodontic RCT protocol to CONSORT reporting standards. This paper gives full details of the protocol and the study for which it was designed.

This patient-centred study has been designed to address a key question in the construction of complete dentures, whether impression material has an impact on subsequent comfort and function of the constructed denture and whether that denture can be adjusted to the satisfaction of the patients. A crossover design has been used to eliminate many of the potentially confounding variables found in denture construction; patients are given 2 sets of dentures and act as their own control. This requires attention to the detail of the duplication techniques for the dentures [16] to ensure the dentures are similar in all aspects apart from the impression material used at the secondary impression stage. In doing this, any preferences expressed can be reliably attributed to the impression material and not any other aspect of the denture construction. Additionally, a habituation period has been included in this study to guard against the potential for bias seen in other crossover studies [9] that patients may prefer the denture they try in the second study period, a bias that has been attributed to the fact that the new dentures can feel very different to the patients existing dentures and the first study period is spent habituating to the new feel of the dentures [9,17]. This study has been blinded to patients, dentists and dental nurses by marking the dentures only with coloured acrylic dots and by ensuring that all adjustment of dentures is managed by a dentist not involved in their construction/fitting. The primary outcome has been chosen to cover both patient-centred considerations (patient preference for denture impression material, previously shown to be a sensitive and effective tool [23,24]) and health-economic considerations (cost-benefits associated with each of the denture impression materials).

The use of complete dentures has a significant impact on the lives of edentulous patients and the quality of secondary denture impressions has the potential to affect both comfort and function of the resultant dentures. There is a dichotomy of opinion between the UK and USA as to which impression material provides the best set of dentures and a paucity of evidence to support either view; this study aims to fill that evidence gap.

The protocol described for this study has potential to be adapted to explore other aspects of denture construction in the future. The use of crossover design, patient preference outcome, attention to detail in denture duplication and the strict blinding and randomisation procedures are designed to deliver robust results and guard against bias.

## Trial status

The first patient was enrolled into IMPROVDENT on the 28^th^ April 2010 and the last patient was recruited on 18^th^ April 2012. The study is being conducted in the Dental Translational and Clinical Research Unit (DenTCRU), Leeds Dental Institute. We expect to report the results in 2013.

Ethical and governance approval for this trial has been obtained from the Leeds West Ethics Committee (ref 09/H1307/106) and the Leeds Teaching Hospitals NHS Trust respectively. The trial progress is monitored by an independent Trial Steering Committee (TSC).

## Abbreviations

ACP: American College of Prosthodontists; AE: Adverse Event; BNF: British National Formulary; CI: Chief Investigator; CRF: Case Report Form; CTRU: Clinical Trials Research Unit; DenTCRU: Dental Translational and Clinical Research Unit; EQ5D EuroQOL: 5 Domains (Quality of Life Health Questionnaire); GCP: Good Clinical Practice; ICH: International Conference on Harmonisation; ICMJE: International Committee of Medical Journal Editors; ISF: Investigator Site File; ITT: Intention To Treat; LDI: Leeds Dental Institute; NHS: National Health Service; NIHR: National Institute of Health Research; OHIP: Oral Health Impact Profile (Questionnaire); OHIP-EDENT: Specialised OHIP for Edentulous people (people with no natural teeth); OHRQoL: Oral Health Related Quality of Life; PI: Principal Investigator; PIS/ICD: Patient Information Sheet/Informed Consent Document; PSSRU: Personal Social Services Research Unit; QOL: Quality of Life; QALY: Quality-Adjusted Life Year; RCT: Randomised Clinical Trial and/or Randomised Controlled Trial; REC: Research Ethics Committee; RU SAE: Related Unexpected Serious Adverse Event; SAE: Serious Adverse Event; SOP: Standard Operating Procedure; TMF: Trial Master File; TMG: Trial Management Group; TSC: Trial Steering Committee.

## Competing interests

The authors declare that they have no competing interests.

## Authors’ contributions

JCG contributed to the overall study design, with particular responsibility for statistical considerations, and led the drafting of this manuscript. SP contributed to the design of the study and leads the Patient and Public Involvement (PPI) aspects of the study. NNC was responsible for the study set-up and overall study coordination. CH participated in the design of the study, with particular responsibility for the health economics. MG contributed to the design of the study, with particular responsibility for the Qualitative research. HLC contributed to the study design and is primarily responsible for the acquisition of clinical data and the clinical interpretation of data. PB contributed to the study design. SB and JW contributed to the design of the statistical analysis plan. SD is responsible for the blinding of the study and for denture construction. GD contributed to the design of the case report forms and is responsible for recruitment of patients, coordination and the acquisition of clinical data. CF is responsible for the coordination of the study. HAC is responsible for data management. SS and CL are PPI representatives and contributed to the design of the study. TPH conceived of the study, and participated in its design and coordination. All authors meet regularly to ensure the smooth running of the study, were involved in the protocol drafting and contributed to and approved the final manuscript.

## Pre-publication history

The pre-publication history for this paper can be accessed here:

http://www.biomedcentral.com/1472-6831/12/37/prepub
